# Knee osteotomies are becoming widely accepted and increasingly used, but rehabilitation techniques still vary widely between surgeons: A survey on current trends

**DOI:** 10.1002/jeo2.70270

**Published:** 2025-05-26

**Authors:** Nicholas I. Kennedy, Nicholas N. DePhillipo, Luke V. Tollefson, Robert F. LaPrade

**Affiliations:** ^1^ Department of Orthopedics Twin Cities Orthopedics Edina Minnesota USA; ^2^ Multicare Orthopedics Northwest Yakima Washington USA; ^3^ Department of Orthopedics University of Pennsylvania Philadelphia Pennsylvania USA

**Keywords:** DFO, HTO, knee osteotomy, PTO, slope correcting osteotomy, survey

## Abstract

**Purpose:**

The purpose of this research survey was to report the current trends regarding the frequency, indications and techniques of knee osteotomies used in sports medicine practices over the past 5 years and during fellowship training.

**Methods:**

Two Institutional Review Board‐exempt anonymous surveys were electronically distributed between May 2023 and August 2023. A 19‐question survey was sent to active members of the American Orthopaedic Society for Sports Medicine and European Society of Sports Traumatology, Knee Surgery and Arthroscopy societies and another 36‐question survey was sent to orthopaedic sports medicine fellows near the end of their training. The surveys included questions regarding type and frequency of osteotomies performed, surgical indications, surgical techniques, preoperative planning and post‐operative weight‐bearing restrictions. Inclusion criteria were those who currently perform knee osteotomy surgery. Descriptive statistics were used for all comparisons.

**Results:**

A total of 100 sports medicine surgeons and 26 sports medicine fellows participated in the survey. The most common type of osteotomy performed was a medial opening‐wedge proximal tibial osteotomy for both surgeons (96%) and fellows (92%). The most used methods to assess coronal and sagittal alignment were long‐leg radiographs (98% for surgeons) and lateral radiographs (66% for surgeons), respectively. Rehabilitation techniques varied between surgeons and fellows and depended on the technique, size of correction and hardware.

**Conclusion:**

Osteotomies are becoming widely accepted as useful techniques to correct bony malalignment. Surgeons are increasing their use of osteotomies, and fellows in training are optimistic about the use of new technologies for osteotomy planning and execution. Trends suggest that osteotomies to correct varus and valgus malalignment for osteoarthritis are the most common, and rehabilitation techniques vary widely between surgeons.

**Level of Evidence:**

Level V.

**Abbreviations**
ACL: anterior cruciate ligament
AOSSM: American Orthopaedic Society for Sports Medicine
DFO: distal femoral osteotomy
ESSKA: European Society of Sports Traumatology, Knee Surgery and Arthroscopy
HTO: high tibial osteotomy
OA: osteoarthritis
PCL: posterior cruciate ligament
PTO: proximal tibial osteotomy
PTS: posterior tibial slope

## INTRODUCTION

Knee osteotomy is a general term used to describe surgical procedures which aim to redistribute load‐bearing forces within the knee joint [16]. The most common types of knee osteotomies are proximal (high) tibial osteotomy (PTO), distal femoral osteotomy (DFO) and slope correcting osteotomies, utilizing both opening and closing wedges, de‐rotational osteotomies and a variety of different surgical and fixation techniques [6, 11, 12, 22, 30, 31]. Indications vary and include knee malalignment in the setting of unicompartmental osteoarthritis (OA) and failed knee ligament reconstructions [15, 18, 19]. While the current literature provides insights into surgical techniques, outcomes and patient selection criteria, there exists a notable gap in research exploring the contemporary practices and future directions of knee osteotomy surgery within sports medicine [5, 9, 19, 20, 27, 28, 29].

Moreover, controversial information exists regarding the factors, like degree of OA, body mass index, and age, influencing surgeons' decision‐making processes when choosing between knee osteotomy and alternative interventions such as partial/total knee arthroplasty [7, 8, 21, 23]. Without a holistic view of these dynamics, clinicians may lack the necessary insights to provide personalized and optimal treatment strategies for their patients. The available literature tends to overlook the perspectives of the surgeons themselves. A surgeon's experience, preferences and perceptions can significantly impact surgical practices. Therefore, exploring the techniques and practice trends of orthopaedic surgeons and fellows in orthopaedic sports medicine training regarding knee joint osteotomy surgery is essential for a comprehensive understanding of the current landscape. Therefore, the purpose of this survey was to report the current trends regarding the frequency, indications, and techniques of knee osteotomies used in sports medicine practices over the past 5 years and during fellowship training.

## METHODS

### Questionnaire development

This study was anonymous and did not meet institutional review board submission criteria and was deemed to be exempt. A 19‐question, anonymous survey was distributed electronically to active members (attending surgeons) of the American Orthopaedic Society for Sports Medicine (AOSSM) and the European Society of Sports Traumatology, Knee Surgery and Arthroscopy (ESSKA) between May 2023 and August 2023. A different, 36‐question, Institutional Review Board‐exempt, anonymous survey was distributed electronically to North American orthopaedic sports medicine fellows near the end of their fellowship training between May 2023 and August 2023. A cover letter that accompanied the questionnaire stated the purpose of the questionnaire and ensured anonymity. The survey included questions regarding practice location (country), experience, type and frequency of osteotomies performed, surgical indications, surgical techniques, preoperative planning and post‐operative weight‐bearing restrictions. Participants were asked to focus on the last 5 years of their practice (for attendings) or their year of fellowship training and subsequent future practice (for fellows) to help identify recent and emerging trends within sports medicine and knee osteotomy procedures. The surveys and the cover letter were created by three authors (NIK, NND and RFL).

Surgeons were identified through two international orthopaedic societies: AOSSM and ESSKA. The survey was sent via email and advertised to active members of AOSSM and ESSKA and targeted orthopaedic surgeons who specialize in sports medicine surgery and osteotomies. All surgeons who currently perform knee osteotomy were allowed to participate in the survey and were only excluded if they reported that they did not perform knee osteotomy surgery. A total of 120 attending sports medicine surgeons were sent the survey. For the sports medicine fellows, all fellows registered within AOSSM were sent the survey and instructed to complete the survey if they participated in osteotomies during their fellowship. All survey participants had the opportunity to decline the questionnaire. Patellofemoral joint osteotomy procedures (tibial tubercle osteotomies or trochleoplasties) were not included.

### Statistical analysis

Data were prospectively collected via an online questionnaire survey tool (SurveyMonkey Inc.). Data were extracted from the online survey database and summarized. Standard descriptive statistics were performed.

## RESULTS

### Demographics, frequency and indications—Attendings

**Figure 1:**
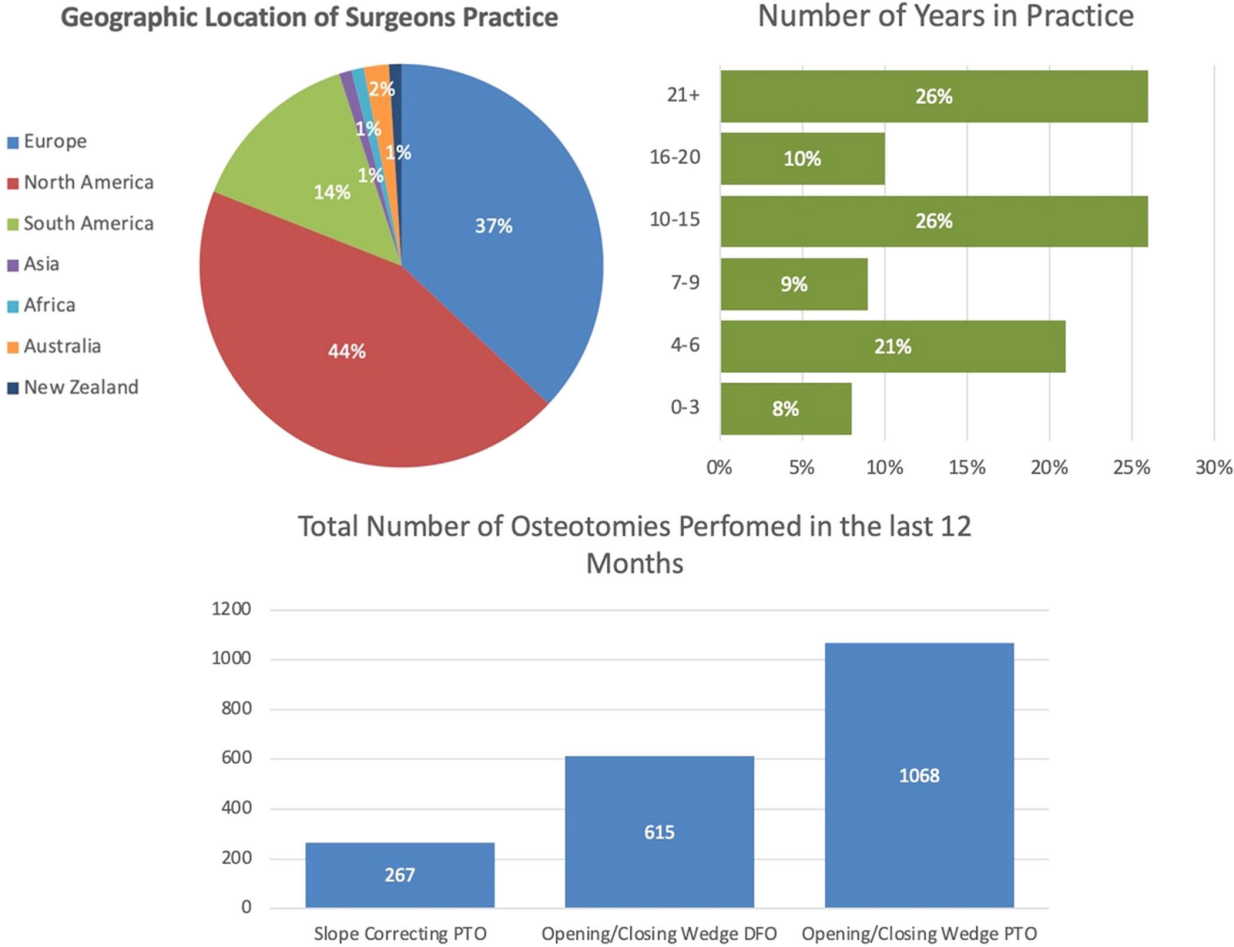
Demographic data of international group of orthopaedic sports medicine surgeons (*n* = 100). DFO, distal femoral osteotomy; PTO, proximal tibial osteotomy.

A total of 120 attending surgeons received the survey, while 100 completed the survey (83.3% completion rate). Of the sports medicine surgeons that participated in the 19‐question survey, the majority (44%) were practising in North America, followed by Europe (37%) and South America (14%). Over half (62%) of the respondents were surgeons practising for over 10 years, with 26% practising for over 21 years and 8% practising for less than 3 years. Over the past 5 years, the respondents collectively reported performing a total of 1068 opening/closing wedge PTOs (average 10.7 surgeries per year), 615 opening/closing wedge DFOs (average 6.1 surgeries per year), and 267 slope correcting tibial osteotomies (average 2.7 surgeries per year) (Figure 1).

All surgeons reported that they routinely assess for coronal alignment in their practice, with the most common assessment reported as utilizing long‐leg standing radiographs (98%), followed by physical examination (73%) (i.e., gait analysis, varus thrust). The most common methods for assessing tibial (sagittal) slope were standard lateral knee radiographs (67%), long leg lateral tibia radiographs (57%) and physical examination (39%) (i.e., genu recurvatum and knee hyperextension). The most common types of osteotomies used in current practices were medial opening‐wedge PTO (96%), opening‐wedge DFO (77%), anterior closing‐wedge PTO (58%), closing‐wedge DFO (54%) and biplanar PTO (52%) The most common indications for knee osteotomy were OA with malalignment (91%), coronal malalignment in the setting of failed prior ligament reconstruction (84%) and increased tibial slope (69%).

In terms of changes in the frequency of knee joint osteotomies over the past 5 years, 40% reported an increase in the overall number of osteotomies performed, 32% reported no significant changes in frequency and 16% reported a decrease in the overall number of osteotomies performed. Overall, 60% of surgeons reported that they began performing more types of knee joint osteotomies and expanded their indications for knee joint osteotomies in the past 5 years.

### Demographics, frequency, indications—Sports medicine fellows

**Figure 2:**
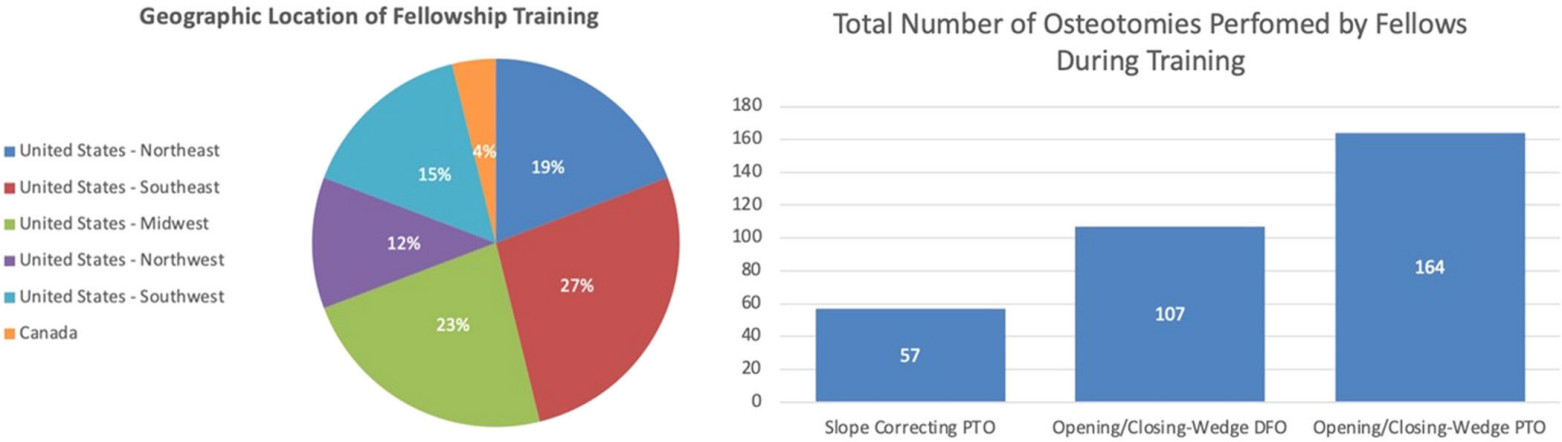
Demographic data of a group of orthopaedic sports medicine fellows during training (*n* = 26). DFO, distal femoral osteotomy; PTO, proximal tibial osteotomy.

A total of 26 orthopaedic sports medicine fellows who performed osteotomies in their fellowships participated in the 36‐question survey; 96% were from the United States (27% Southeast, 23% Midwest, 19% Northeast, 15% Southwest and 12% Northwest), and 4% (one) were from Canada. During their training, the respondents collectively reported performing a total of 164 opening/closing wedge PTOs (average 6.3 surgeries per year), 107 opening/closing wedge DFOs (average 4.1 surgeries per year), and 57 slope correcting tibial osteotomies (average 2.2 surgeries per year). The fellowship survey was split into two sections, one asking about what they did as a fellow and the other about what they plan on doing as an attending (Figure 2).

During their training, all but one fellow (4%) reported that they routinely assess for coronal alignment in their training, with the most common assessment reported as long‐leg standing radiographs (96%), followed by visual inspection examination (65%). The most common methods in training for assessing tibial (sagittal) slope were standard lateral knee radiographs (69%), long leg lateral tibia radiographs (50%), and physical examination (42%) (i.e., genu recurvatum and knee hyperextension). The most common types of osteotomies performed in training were medial opening‐wedge PTO (92%), opening‐wedge DFO (69%), anterior closing‐wedge PTO (42%), biplanar PTO (35%) and closing‐wedge DFO (31%). The most common indications for knee osteotomy were OA with malalignment (73%), coronal malalignment in the setting of failed prior ligament reconstruction (65%) and increased tibial slope (46%).

For their future practice, all fellows reported that they will routinely assess for coronal alignment, with the most common anticipated assessment reported as long‐leg standing radiographs (100%), followed by visual inspection examination (69%). The most common anticipated methods for assessing tibial (sagittal) slope in their practice were standard lateral knee radiographs (65%), long leg lateral tibia radiographs (62%) and physical examination (58%) (i.e., genu recurvatum and knee hyperextension). The most common types of osteotomies that fellows intended to incorporate into their future practice were medial opening‐wedge PTO (92%), opening‐wedge DFO (73%), anterior closing‐wedge PTO (62%), closing‐wedge DFO (46%) and biplanar PTO (38%). The most common indications they anticipated using for knee osteotomies were OA with malalignment (85%), coronal malalignment in the setting of failed prior ligament reconstruction (85%) and increased tibial slope (73%).

### Techniques and standards of practice—Attendings

For the indication of knee OA with malalignment, the majority (53%) reported using the apex of the lateral tibial eminence as the primary landmark for the new weight‐bearing axis for valgus‐producing knee osteotomies. In addition, 46% reported using the centre of the joint as a landmark for varus‐producing knee osteotomies. For indications other than OA with malalignment, 54% reported using the centre of the joint to determine the new weight‐bearing axis.

**Figure 3:**
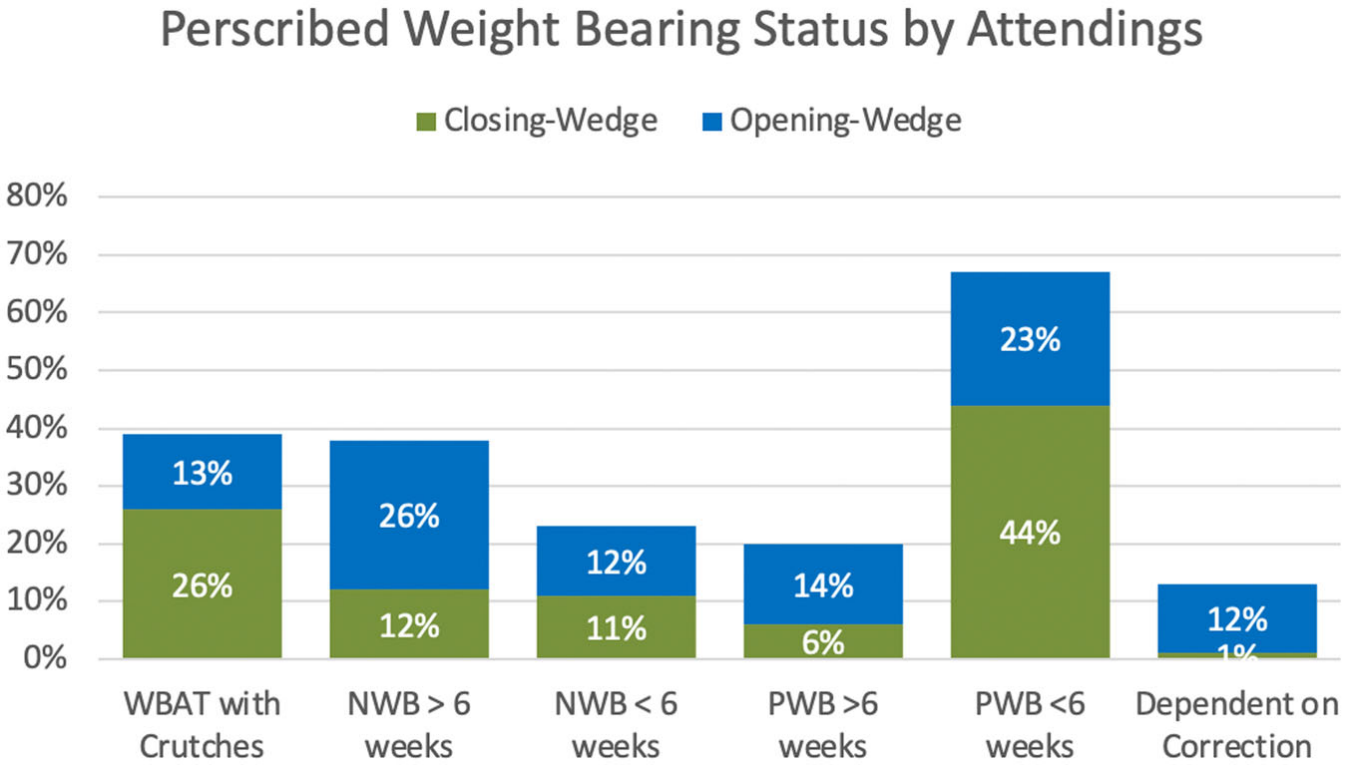
Prescribed weight‐bearing regimen following opening‐ and closing‐wedge osteotomies for attendings. NWB, non‐weight‐bearing; PWB, partial weight‐bearing; WBAT, weight‐bearing as tolerated.

The prescribed weight‐bearing regimen following opening‐ and closing‐wedge osteotomies is reported in Figure 3. Overall, the most common weight‐bearing regimen reported by surgeons was partial weight bearing for less than 6 weeks (44% for closing‐wedge osteotomies and 23% for opening‐wedge osteotomies). Regarding the utilization of an unloader brace for knee osteotomy patients, 74% of surgeons reported using an unloader brace in some capacity (preoperatively [to screen for the potential effectiveness of an osteotomy] and post‐operatively) and 24% reported no use of an unloader brace.

### Techniques and standards—Fellows

#### During training

For the indication of medial compartment knee OA with malalignment, the majority (62%) reported using the apex of the lateral tibial eminence as the landmark for the new weight‐bearing axis correction for valgus‐producing knee osteotomies. In addition, for the indication of lateral compartment knee OA with malalignment, the majority (58%) reported using the apex of the medial tibial eminence as the landmark for varus‐producing knee osteotomies. For other indications than OA with malalignment, 38% reported using the centre of the joint to determine the new weight‐bearing axis.

**Figure 4:**
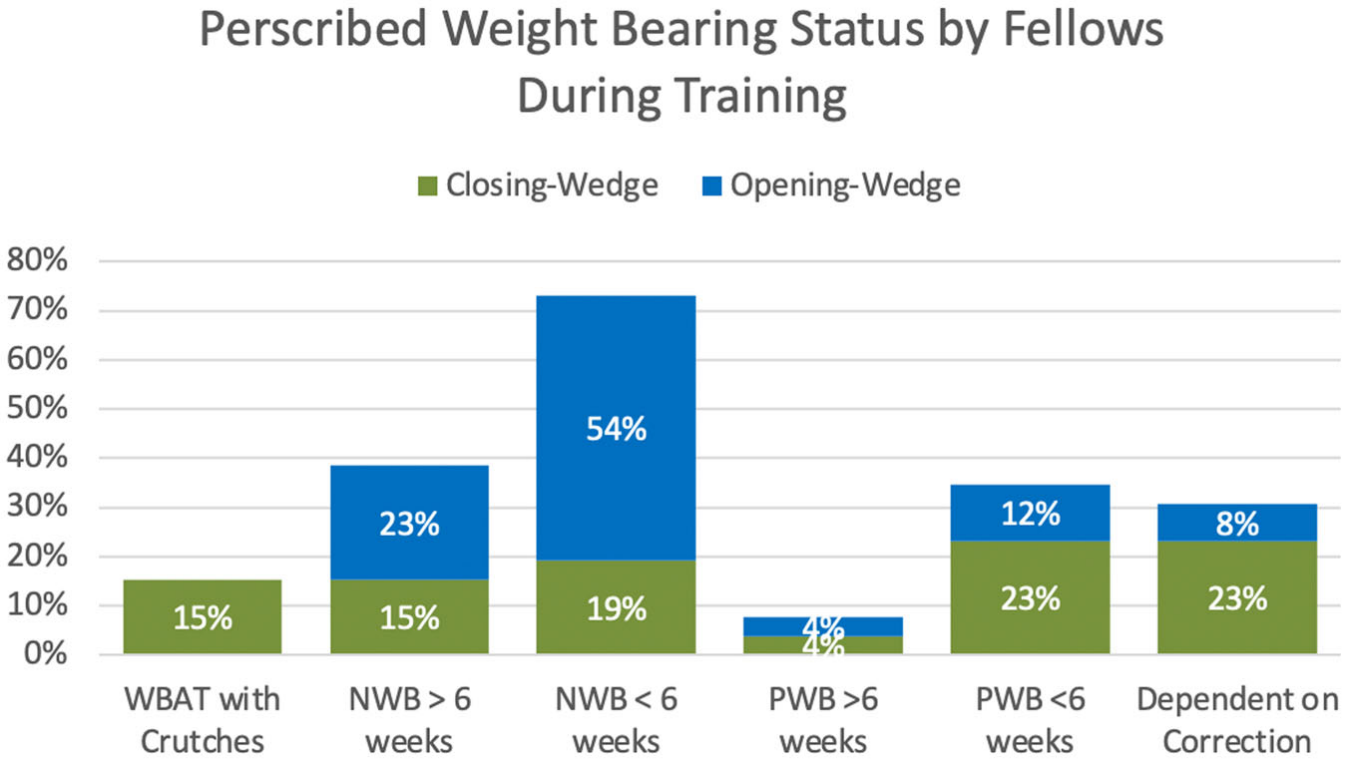
Prescribed weightbearing regimen following opening‐ and closing‐wedge osteotomies of distal femoral and proximal tibial reported by fellows during their training. NWB, non‐weight‐bearing; PWB, partial weight‐bearing; WBAT, weight‐bearing as tolerated.

The prescribed weight‐bearing regimen following opening‐ and closing‐wedge osteotomies is reported in Figure 4. For closing wedge osteotomies, the most common weight‐bearing regimen reported by fellows during training was partial weight bearing for less than 6 weeks and weight bearing dependent on the magnitude of correction, 23% each. For opening‐wedge osteotomies, the most common weight‐bearing protocol was non‐weight bearing for <6 weeks at 54% of respondents. Regarding the utilization of an unloader brace for knee osteotomy patients, 81% of fellows in training reported using an unloader brace in some capacity (preoperatively [for screening for the effectiveness of a potential osteotomy] and post‐operatively) and 19% reported no use of an unloader brace.

#### Future practice

For the indication of medial knee OA with malalignment, the majority (62%) reported they would use the apex of the lateral tibial eminence as the new weight‐bearing axis correction landmark for valgus‐producing knee osteotomies. In addition, for the indication of lateral knee OA with malalignment, the majority (58%) reported they would use the apex of the medial tibial eminence as the landmark for varus‐producing knee osteotomies. For indications other than OA with malalignment, 38% reported they would use the centre of the joint to determine the new weight‐bearing axis.

**Figure 5:**
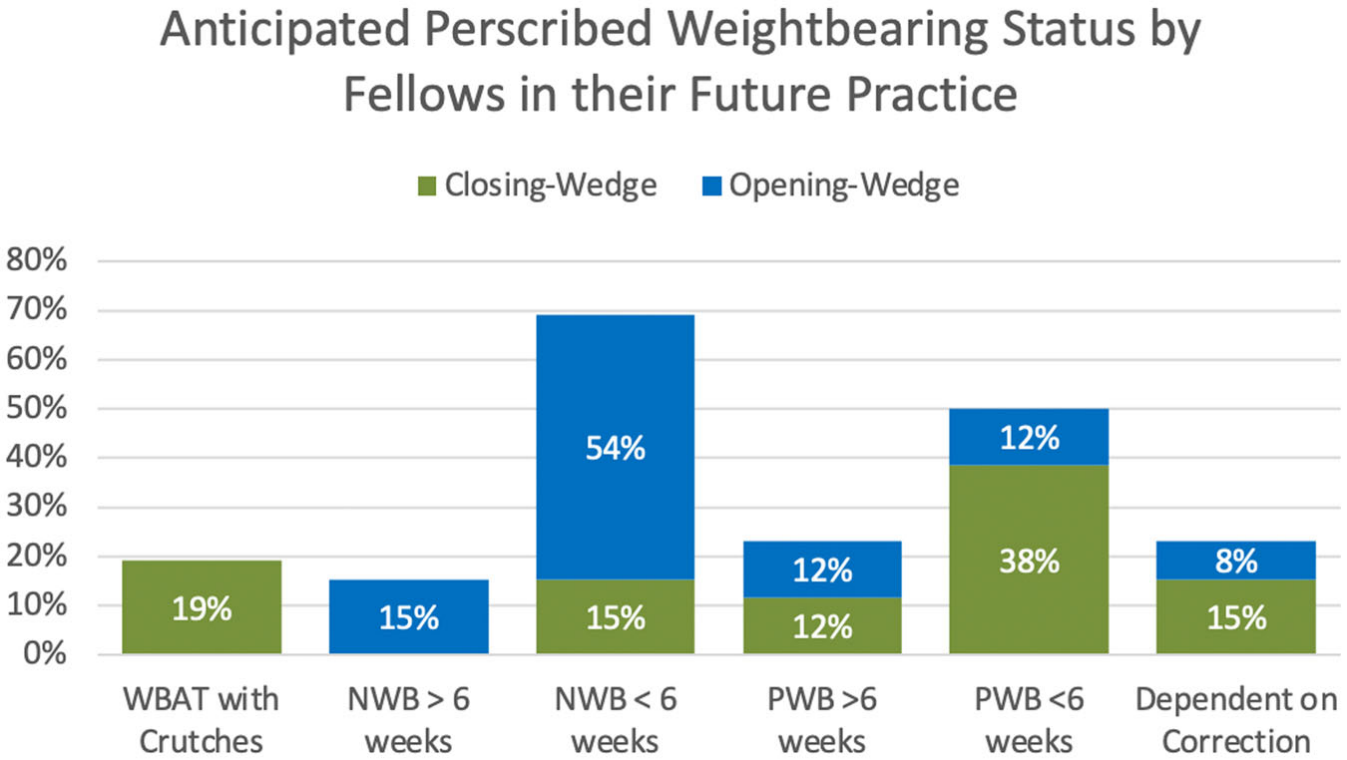
Anticipated prescribed weight‐bearing regimen following opening‐ and closing‐wedge osteotomies of distal femoral and proximal tibial for fellows during their future practice. NWB, non‐weight‐bearing; PWB, partial weight‐bearing; WBAT, weight‐bearing as tolerated.

For closing wedge osteotomies, the most common anticipated weight‐bearing regimen reported by fellows for their future practice was partial weight bearing for less than 6 weeks (38%). For opening‐wedge osteotomies, the most common anticipated weight‐bearing protocol was non‐weight bearing for <6 weeks with 54% of respondents (Figure 5). Regarding the utilization of an unloader brace for knee osteotomy patients, 96% of fellows anticipated using an unloader brace in some capacity (preoperatively and post‐operatively), and 4% anticipated not using an unloader brace. Additionally, 27% were very likely and 38% were likely to use navigational assistance or patient‐specific instrumentation for their future osteotomy practice, with most opting not to use this technology due to a lack of familiarity/exposure (44%) or cost (39%).

## DISCUSSION

The most important finding of this study was that over the past 5 years, 60% of surgeons in our cohort began performing more types of knee osteotomies and expanded their indications for knee osteotomies, and 40% reported an increase in an overall number of osteotomies performed in that same time period. This study also found that opening/closing wedge PTOs were the most performed knee joint osteotomy, followed by opening/closing wedge DFOs, and finally slope correcting PTOs. In addition, long leg radiographs were the most commonly reported method to assess coronal alignment, and standard lateral radiographs were the most commonly reported method to assess sagittal alignment. Furthermore, there were differences in weight‐bearing protocols following opening and closing wedge osteotomies, highlighting the need for future research for optimization and standardization of post‐operative protocols following knee osteotomy.

In the current study, a survey‐based research approach was employed to gain insights directly from orthopaedic sports medicine surgeons and orthopaedic sports medicine fellows regarding knee joint osteotomies. This approach offers several advantages, including the ability to capture real‐world practices, emerging trends, and the reasoning behind surgeons' decision‐making processes. Our survey found that 60% of these surgeons in practice had expanded their indications for and were performing more types of knee osteotomies, while 40% reported an increase in overall volume. This can likely be explained, in part, by newer studies highlighting the ability to perform osteotomies in patients with more advanced OA, higher BMIs, and increased age with satisfactory patient outcomes [8, 21, 23]. This is important because it marks a paradigm shift in the field of orthopaedic sports medicine. With the invention and advancement of total knee arthroplasty, osteotomy usage dropped precipitously and indications shrank from 1990 until recently. However, more recently, as our understanding of the importance of osseous alignment on proper knee biomechanics has expanded, there has been a renewed interest in osteotomy procedures, which has been evident with the increased focus in the recent literature. These survey findings not only demonstrate increased usage amongst tenured surgeons, but further, the responses of the fellows demonstrate their exposure to osteotomies in training, all of which help to quantify the degree or extent of osteotomy usage in current practice.

Regarding the types of osteotomies currently being performed, the order was identical for current orthopaedic sports medicine surgeons and orthopaedic sports medicine fellows alike: opening/closing wedge PTOs, followed by opening/closing wedge DFOs and slope‐correcting PTOs. Osteotomies are becoming more common for treating OA due to malalignment, to prevent increased strain on meniscal or ligament repair/reconstructions, to offload chondral procedures, or as a staged procedure during ligamentous reconstruction [1, 15, 18, 27].

For assessing the degree of malalignment and what type of correction should be performed, long leg radiographs for coronal alignment and standard lateral radiographs for sagittal alignment were the most common methods [10, 14, 26]. Coronal alignment can determine the degree of varus or valgus malalignment and can also determine if the deformity is originating from the femur or the tibia [13]. It is important to note that it has been reported that a standard AP radiograph is not sufficiently accurate to utilize for assessing a patient's weight‐bearing alignment [14]. For sagittal alignment, increased posterior tibial slope (PTS) puts more stress on the anterior cruciate ligament (ACL) or ACL graft, and decreased PTS puts more stress on the posterior cruciate ligament (PCL) or PCL graft [3, 4]. The degree of correction can be determined with standard lateral radiographs to attempt to correct the slope to a normal value [10].

This study found differences in weight‐bearing protocols for opening and closing wedge osteotomies. Following any type of osteotomy, proper rehabilitation techniques should be followed to allow for proper bone healing and prevent malunions or non‐unions. For opening wedge proximal tibial osteotomies, a recent systematic review by van Haeringen et al. [33] reported that an early full weight bearing protocol may be used, but only in cases of small corrections and with no hinge fractures. However, for closing wedge osteotomies or slope correction osteotomies (opening or closing), there is a lack of literature reporting on rehabilitation techniques and outcomes for patients. This helps explain the discrepancies in the reported weight‐bearing protocols for the surgeons and fellows participating in this survey. Depending on if they use staples or plates and screws for fixation, the types of fixation plates, the size of the correction, if the cortical hinge fractured, or the type of osteotomy, the rehabilitation technique may be up to the discretion of the physician [24, 32]. This highlights the importance of more research into the optimal post‐operative rehabilitation protocols for patients to not only reduce the risk of complications, but also to improve patient outcomes and optimize their return to activities.

Another interesting finding from this survey was the different attitudes towards osteotomies between attendings and fellows. The biggest difference came in the form of which indications the fellows will perform osteotomies in their future practice, compared to how they were taught. For example, the fellows plan to incorporate osteotomies for coronal malalignment for failed ligament reconstructions (73% vs. 46%), increased tibial slope (85% vs. 65%), and decreased tibial slope (31% vs. 15%) at a much higher rate than they were taught. Furthermore, the fellows plan to use unloader braces at a higher rate for both preoperative screening (92% vs. 77%) and in the post‐operative phase (31% vs. 12%) compared to how they were taught. This finding also alludes to the overall trends in osteotomy usage with increasing usage almost across the board, especially in younger physicians.

In addition to elucidating current practices, the survey probed into the anticipated future directions of knee osteotomy surgery. A substantial proportion of the fellowship respondents expressed optimism about their intended integration of advanced technologies, such as patient‐specific instrumentation and computer‐assisted navigation, into their practices [2, 17, 25]. These technologies have the potential to enhance surgical precision and outcomes, further establishing knee osteotomy surgery as a viable procedure for joint preservation [2, 34]. Furthermore, the survey sheds light on the evolving attitudes towards knee osteotomy surgery. While the procedure has historically been underutilized, especially in comparison to partial and total knee arthroplasty, the survey results suggest a growing interest in knee preservation strategies among surgeons. This shift can be attributed to increasing evidence supporting the benefits of knee osteotomy, coupled with patients' preference for joint‐preserving options.

There were some limitations inherent in this study, such as the use of a survey questionnaire. First, the subjective reports and common trends of the survey respondents cannot be validated with evidence‐based recommendations. Second, since this study design relied on self‐reported data from surgeons, there is potential for response bias. This could result in variability in the reported survey responses. Finally, the sample size, while primarily composed of experienced AOSSM and ESSKA knee surgeons, may not fully represent global practice patterns, and the study does not account for factors such as surgical skill or institutional protocols that may influence technique selection. Further research is needed to correlate current clinical practices with patient outcomes, helping to establish evidence‐based guidelines for knee osteotomies.

## CONCLUSION

Osteotomies are becoming widely accepted as useful techniques to correct bony malalignment. Surgeons are increasing their use and indications of osteotomies, and fellows in training are optimistic about the use of new technologies for osteotomy planning and execution. Trends suggest that osteotomies to correct varus and valgus malalignment for OA are the most common, and rehabilitation techniques vary widely between surgeons.

## AUTHOR CONTRIBUTIONS

Conception: Nicholas N. DePhillipo, Nicholas I. Kennedy and Robert F. LaPrade. Design of the work: Nicholas N. DePhillipo, Nicholas I. Kennedy and Robert F. LaPrade. Interpretation of data: Nicholas N. DePhillipo, Nicholas I. Kennedy and Luke V. Tollefson. Substantial edits to work: All authors. Approved the submitted version: All authors. Agreed to be accountable for work: All authors.

## CONFLICT OF INTEREST STATEMENT

Robert F. LaPrade is a consultant for Ossur and Smith and Nephew, receives royalties from Ossur, Smith and Nephew and Elsevier, and receives research grants from AANA, AOSSM, Ossur, Arthrex and Smith and Nephew. Nicholas I. Kennedy is a consultant for Smith & Nephew, Vericel, Enovis and CTM Biomedical. The remaining authors declare no conflicts of interest.

## ETHICS STATEMENT

Ethics approval and consent to participate, the institutional review board noted an exempt status for this anonymous survey.

## Data Availability

Data Availability Statement is not available.
